# Vasopressin and Oxytocin Reduce Food Sharing Behavior in Male, but Not Female Marmosets in Family Groups

**DOI:** 10.3389/fendo.2017.00181

**Published:** 2017-07-27

**Authors:** Jack H. Taylor, Allison A. Intorre, Jeffrey A. French

**Affiliations:** ^1^Department of Psychology, University of Nebraska at Omaha, Omaha, NE, United States; ^2^Callitrichid Research Center, Omaha, NE, United States; ^3^Department of Biology, University of Nebraska at Omaha, Omaha, NE, United States

**Keywords:** food sharing, provisioning, oxytocin, vasopressin, marmoset, sibling, parental care

## Abstract

Oxytocin (OT) is critical for lactation and maternal care, but OT and the related nonapeptide vasopressin are important for caregiving behaviors in fathers and alloparents as well. This experiment tested the effects of vasopressin and OT on food sharing in marmoset families. We treated caregivers (parents, siblings) with intranasal vasopressin, OT, or saline, and then paired them with the youngest marmoset in the family. Caregivers were given preferred food, and then observed for food sharing and aggressive behavior with young marmosets. OT reduced food sharing from male alloparents to youngest siblings, and fathers that received vasopressin refused to share food with their youngest offspring more often than when treated with OT. Vasopressin increased aggressive vocalizations directed toward potential food recipients in all classes of caregivers. These results indicate that vasopressin and OT do not always enhance prosocial behavior: modulation of food sharing depends on both sex and parental status.

## Introduction

In mammals, mothers begin providing nutritional support (i.e., lactation) immediately after the delivery of offspring, and this process is regulated by the nonapeptide hormone oxytocin (OT) and its cognate receptor (1). OT is also an important modulator of other maternal behaviors in addition to lactation, as demonstrated in multiple experimental approaches. OT administered intracerebroventricularly (i.c.v.) induces maternal behavior in estrogen-primed rats (2), and OT receptor (OTR) antagonists administered directly into the ventral tegmental area, or administered directly into the medial preoptic area block the normal expression of postpartum maternal behavior in rats (3, 4), indicating a causal role for OT in the onset of maternal behavior. Arginine vasopressin (AVP), a nonapeptide that is closely related to OT, also modulates maternal behavior. AVP and OT are highly similar nonapeptides, differing at only two amino acid positions, and each can bind and activate the others’ receptors [reviewed in Ref. (5, 6)], but often AVP and OT affect different behavioral patterns associated with mother–offspring interactions. Pharmacological manipulations of AVP in the brain indicate that AVP is an important neuromodulator of “active” maternal behavior, including the enhancement of defensive aggression [(3, 4); c.f. (7, 8)]. Data from correlational studies investigating OT or AVP support a role for both nonapeptides in the regulation of maternal care (9–11), though there is some concern over whether peripheral measures of nonapeptides accurately reflect levels in the central nervous system (12, 13). These experimental and correlational data show that OT and AVP are important neuromodulators of maternal behavior.

There is strong evidence that OT modulates behavior in caregivers other than the mother, including fathers (paternal care), as well as older siblings and unrelated, reproductively inexperienced males and females (alloparental care). OT-like compounds facilitate male parental care in several non-mammalian species [(14, 15); c.f. (16)]. OT induces maternal-like behavior in female sheep exposed to unrelated offspring and enhances pup care in reproductively naïve female rats, animals which would not otherwise provide care spontaneously (2, 17, 18). Moreover, OTR knockdown reduces spontaneous alloparental behavior in female prairie voles (19). Male caregiving behavior is affected by OT as well; i.c.v. OT enhances food provisioning behavior in marmoset monkey fathers (20), and intranasal OT increases responsiveness to infant stimuli in marmoset males (21). In humans, intranasal OT in fathers enhances infant touching and joint father–infant social gaze (22). Correlational data support these pharmacological studies in fathers and alloparents. In general, OT-system activity, measured both peripherally and centrally, increases with caregiving behavior in human fathers (9, 11, 23), non-human alloparents (24, 25), and fathers of biparental non-human species (24, 26–29). Taken together, these data show that OT is important for modulating the behavior of all caregivers.

Arginine vasopressin and its non-mammalian analogs also affect caregiving behavior in fathers and alloparents. In reproductively inexperienced male prairie voles, i.c.v.-administered AVP enhanced, and a V1aR antagonist inhibited, alloparental behavior (30). Similarly, AVP enhanced responsiveness to infant stimuli in female marmosets [including infant-naïve females (21)]. Correlational data also suggest that AVP-system activity enhances parental behavior. Exposure to young enhances AVP-system activity in the brain (27, 31–34), and enhanced paternal behavior is positively associated with AVP-system activity (9, 35). In other species and contexts though, AVP activity inhibits caregiving behavior by non-mothers. AVP administration reduced nest building in biparental old-field mouse fathers, and inhibition of AVP neuron activity enhances nest building in male and female laboratory mice (36). Similarly, V1aR mRNA is downregulated in biparental California mouse fathers, and increased V1aR mRNA expression in California mice is associated with longer latencies to approach pups (28). AVP-mediated inhibition of paternal behavior is present in non-mammals as well; intraperitoneal vasotocin inhibited paternal behavior in poison frogs and clownfish (14, 16). In other contexts, the relationship between AVP and caregiving behavior by non-mothers is less clear. AVP administration did not affect responsiveness to infant stimuli in male marmosets (21), and V1aR antagonist treatment in reproductively inexperienced male prairie voles was only effective at reducing alloparental behavior when it was co-administered with an OTR antagonist (37). Thus, the relationship between AVP and caregiving in non-mothers is less clear than the relationship between OT and caregiving in non-mothers, and it is less clear than the relationship between AVP and caregiving behavior in mothers.

Females are the primary provisioners early in mammalian development (*via* lactation) but in marmosets, mothers, fathers, and alloparents participate in food sharing behavior to infants both during and after weaning. Moreover, the relationship between increased urinary OT and food provisioning in marmosets strengthens during and after weaning (24). To date, only one study has shown that OT manipulation enhances food sharing behavior. Saito and Nakamura (20) treated marmoset fathers with i.c.v. OT and found that OT reduced food sharing refusals to young, a measure of enhanced food provisioning, but not older offspring. OT did not affect active food sharing in fathers, though. We sought to expand Saito and Nakamura’s findings by investigating both OT- and AVP-mediated food sharing in all family members. In this experiment, we investigated the influence of AVP and OT on food sharing with juvenile family members by fathers, mothers, and older siblings (alloparents) in marmosets. We treated marmoset mothers, fathers, and alloparents with intransal AVP, OT, or saline control, and then tested their provisioning of rewards in a food sharing paradigm. In this paradigm, caregivers could choose to share or withhold preferred food items with the youngest member of the family. If AVP and OT affect food provisioning *via* general prosocial mechanisms, then we would expect both AVP and OT to increase food provisioning in all caregivers, regardless of sex or parental status (breeder vs. alloparent). Alternatively, if AVP and OT act *via* mechanisms specific to sex or parental status of food provisioners, then we would expect differential rates of food provisioning between AVP- and OT-treated mothers, fathers, and alloparents. Because AVP enhanced responsiveness to infant stimuli in marmoset females and OT enhanced responsiveness to infant stimuli in marmoset males (21), we expected a similar pattern with respect to food provisioning; we expected AVP to enhance food sharing behavior in mothers and female alloparents and OT to enhance food sharing behavior in fathers and male alloparents.

## Materials and Methods

### Subjects

We used 17 marmosets (*Callithrix jacchus*) from three different family groups at the University of Nebraska at Omaha’s Callitrichid Research Center as subjects. Twelve served as potential food provisioners (four adult parents and eight older sibling alloparents, ages 1.15–6.7 years) and five were juvenile marmosets (30–60 weeks of age) that served as potential food recipients. Breeding females were contracepted with cloprostenol (38) to prevent the confounding effects of the presence of nursing and dependent infants within family groups. Thus, all potential food recipients were the youngest animals in their family groups. Table 1 provides demographic and social information on the animals included in the experiment. Marmosets were housed in large family enclosures (1.0 m × 2.5 m × 2 m), and each enclosure had two smaller holding areas (30 cm × 30 cm × 66 cm each) in which all food sharing trials occurred. Marmosets were fed a daily diet of commercial marmoset diet (Science Diet), at approximately 0900 h, and fresh fruits, eggs, mealworms, and yogurt, at approximately 1500 h. Further details on colony management and husbandry can be found in Ref. (39). All procedures were approved by the University of Nebraska at Omaha/University of Nebraska Medical Center IACUC (#15-005-04-FC).

**Table 1:** Marmoset family demographics and recipient pairings.

Family ID	Parents (ages)	Alloparent siblings (ages)	Recipient juveniles (sex/age)
C1	Mother (5.7 years)		Juvenile 1 (F/0.7 years)
	Father (6.0 years)		Juvenile 1
C2	Mother (6.7 years)	Male 1 (2.1 years)	Juvenile 1 (F/0.7 years)
	Father (6.7 years)	Female 1 (2.1 years)	Juvenile 2 (F/0.7 years)
		Female 2 (1.2 years)	Juvenile 1
		Male 2 (1.2 years)	Juvenile 2
C3	Mother^a^ (5.7 years)	Female 1 (2.0 years)	Juvenile 1 (M/1.1 years)
	Father^a^ (3.4 years)	Female 2 (2.0 years)	Juvenile 2 (M/1.1 years)
		Male 1 (1.6 years)	Juvenile 1
		Male 2 (1.6 years)	Juvenile 2

*^a^Indicates these animals were removed from the study because they refused experimenter-provided food*.

### Identification of Preferred Food Items

We wanted to identify foods that were preferred enough by marmosets to elicit consistent food begging by juveniles, but not so highly preferred that provisioners would refuse to share them. We surveyed our colony (four males, six females from Table 1, plus an additional male and an additional female) to identify preferred food items using a two-choice food preference test (40). The food items tested were Science marmoset diet, breakfast cereal (Honey Nut Cheerios©), apple, and marshmallows. Adult and subadult marmosets were presented with two food items on a tray, separated by 2.5 cm, and we recorded which food item was selected first among each food pair. All possible combinations of food item pairs were presented to each marmoset at least four times, with order of testing food pairs randomized and position of food items on the tray alternated between trials.

### Food Sharing Test

At the beginning of each session, the marmoset serving as food provisioner was briefly manually restrained and treated intranasally with either the variant of OT native to marmosets (Pro^8^-OT; approximately 150 µg/kg), vasopressin (approximately 133 µg/kg; ~80 IU), or a saline control. Intranasal treatments were applied dropwise in a volume of 50 µL per nostril. These doses have been shown to alter social behavior in marmosets and Titi monkeys (21, 41–43). Each provisioner was exposed to all three treatments in a counterbalanced order, with at least 48 h between treatments. Salivary OT in humans returns to near baseline levels in less than 7 h after intranasal administration (44). The marmoset was returned to the home cage, and a period of 20 min was given to allow uptake of the treatment (45, 46). After 20 min, the provisioner and recipient were moved to a holding area within the home cage, eliminating the potential for other family members to interfere with potential food provisioning. The provisioner and the recipient were briefly separated with a slotted barrier, and the provisioner was offered a piece of food in a dish. As soon as the provisioner obtained the food item, we removed the barrier, and interactions between the caregiver and recipient were recorded by a single observer who was blind to experimental treatment condition for the provisioner.

Specific behaviors of interest were begging, food sharing, food sharing refusals, and vocalizations. *Begging (count)* was recorded when the recipient marmoset made contact with the provisioner when attempting to take the apple or cereal. *Food sharing (count, latency)* was recorded when the provisioner transferred or allowed recipient to take part or all of the food provided. *Food sharing refusals (count)* were recorded when a beg occurred, but sharing did not. *Begging cries (count)* from the recipient and *aggressive “Ehr-Ehr” vocalizations (count)* by the provisioner were also recorded for each trial. To account for trial-by-trial differences in recipient behavior, we recorded if the recipient *did not see (yes/no)* food before it was eaten, recipient appeared to see food, but had *no interest (yes/no)*, and recipient *watched (yes/no)* caregiver eat food, but did not attempt to take food.

Each session of testing consisted of 20 1-min trials, and apple and cereal were alternated in successive trials. If the provisioner dropped the piece of food before the barrier between the provisioner and recipient was removed, an additional food item was given to the provisioner. Each provisioner:recipient pair was tested under all three experimental conditions (OT, AVP, saline).

### Data Analysis

We used a trial-by-trial analysis to evaluate effects of treatment, sex, and caregiver parental status within the family (parent vs. alloparent). We used a Linear Mixed Model analysis, and nested food sharing trials within testing sessions, sessions within individual marmosets, and marmosets within families. This strategy allowed us to control for trial-by-trial differences in recipient and provisioner hunger status, motivation, or attention, as well as experiment-wide differences in recipient age and family size. Moreover, we were able to appropriately treat families, individuals, and testing sessions as non-independent entities. Our final model is described in Eq. 1. Significant main effects and interactions were explored using Fisher’s *post hoc* tests, using a Satterthwaite approximation for degrees of freedom.

$$\begin{array}{ll} \mathrm{Behaviour}= & \mathrm{Caregiver Sex}\times \mathrm{Treatment}\times \mathrm{Parental Status}\times \mathrm{Food Type}\\ \; & \;+\mathrm{Family Size}+\mathrm{Recipient Age}+\mathrm{Session Number}\\ \; & \;+\mathrm{Trial Number}+\mathrm{Recipient}{\text{ Interest}}^{a}\\ \; & \;+\mathrm{error}(\mathrm{FamilyID})+\mathrm{error}(\mathrm{MonkeyID})\\ \; & \;+\mathrm{error}(\mathrm{SessionID})+\mathrm{error}(\mathrm{residual})\;(1) \end{array}$$

Equation 1. Template model for analysis of behavioral data. Bolded variables indicate primary tests of hypotheses. ^a^*Recipient Interest* was composed of three separate variables and corresponding regression coefficients: recipient did not see food before it was eaten, recipient appeared to see food, but had no interest, and recipient watched caregiver eat food, but did not attempt to take food.

## Results

### Food Preference

Adult marmosets showed a clear hierarchical preference profile for the four food items we tested. Standard diet was never preferred over other foods, and marshmallows were always preferred over other foods. However, there was no overall preference for apples vs. cereal (Table 2, bolded), thus apples and cereal were intermediate in preference compared to diet and marshmallow. In order to maximize food begging while optimizing rates of food sharing (i.e., prevent floor or ceiling effects due to food preference), we chose apples and cereal as our food items in our food sharing test.

**Table 2:** Choice matrix for all food items paired with all other food items.

	Chosen food (%)			
Paired food	Diet	Apple	Cereal	Marshmallow
Diet	–	100^a^	97.9^a^	100^a^
Apple		–	**62.5**	70.8^a^
Cereal			–	70.8^a^
Marshmallow				–

*Bold values indicate no significant preference for chosen food over paired food*.

*^a^Indicate percentage for chosen food was significantly different from 50% [*t*(11) > 2.41, *p* < 0.05]*.

### Food Sharing Test

**Figure 1:**
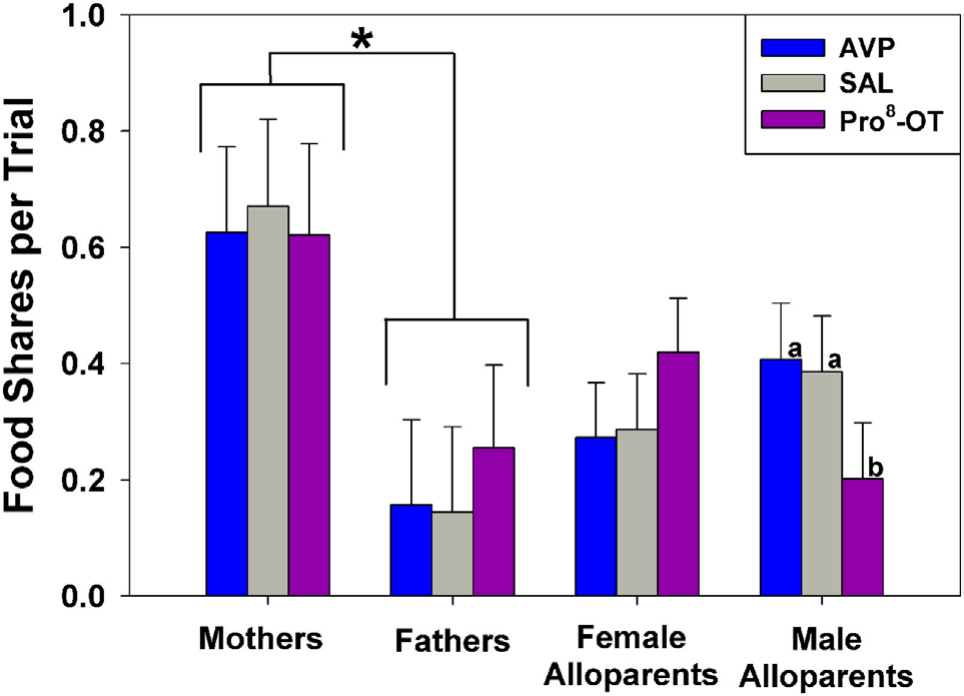
Food sharing from caregivers (parents, alloparents) to the youngest family members. Mothers shared significantly more than fathers did. Male alloparents treated with Pro^8^-oxytocin (OT) shared less than when they were treated with saline or arginine vasopressin (AVP). Asterisks indicate significant differences between social roles using a Fisher’s *post hoc* test (*p* < 0.05). Bars with differing letters indicate significant differences across treatments within individuals using a Fisher’s *post hoc* test.

**Figure 2:**
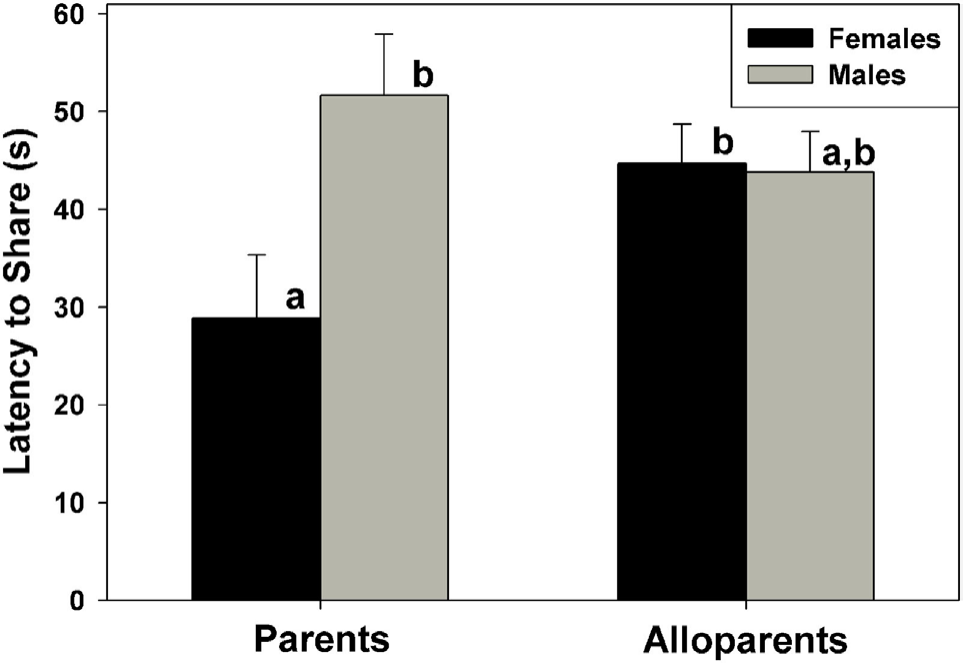
Latency for caregivers (parents, alloparents) to share food to the youngest family members. Mothers shared significantly faster than fathers and female alloparents. Bars with differing letters indicate significant differences between social roles using a Fisher’s *post hoc* test.

Food sharing was associated with the parental status of the provisioner, and it was affected by the interaction between parental status and nonapeptide treatment. Mothers shared more often than fathers, but otherwise there were no differences in rates of food sharing among parents or alloparents [Figure 1; *F*_(1, 13.65)_ = 6.23, *p* = 0.026]. Mothers also had shorter latencies to share food than fathers and female alloparents [Figure 2; *F*_(1, 13.7)_ = 7.28, *p* = 0.018]. Male alloparents were the only family members whose rates of food sharing were altered by nonapeptide treatment. In male alloparents, Pro^8^-OT reduced food sharing compared to AVP and saline [*F*_(2, 26.3)_ = 3.45, *p* = 0.047], but neither Pro^8^-OT nor AVP changed rates of food sharing in mothers, fathers, or female alloparents nor did it affect latencies to share. Provisioners shared marginally, but not significantly, more often [*F*_(1, 11.29)_ = 3.78, *p* = 0.07; Table S1 in Supplementary Material] and faster to younger recipients than to older recipients [*F*_(1, 11.31)_ = 4.31, *p* = 0.06, Table S2 in Supplementary Material].

**Figure 3:**
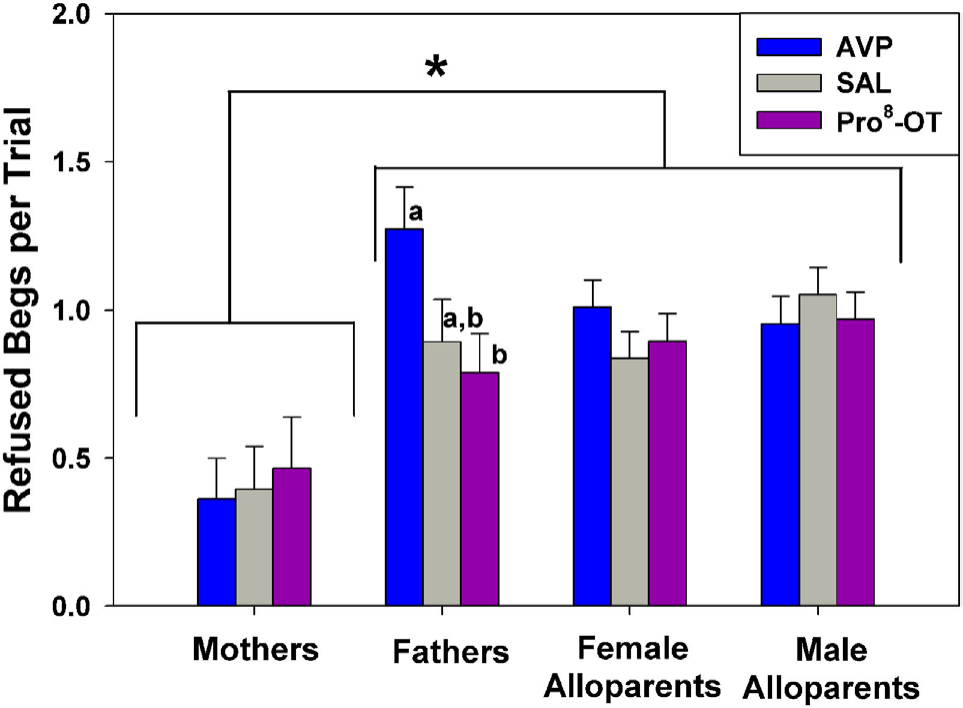
Caregiver (parents, alloparents) food share refusals after a recipient food beg. Mothers refused to share food less often than any other caregiver group. Fathers treated with arginine vasopressin (AVP) refused more when treated with AVP compared to Pro^8^-oxytocin (OT). Asterisks indicate significant differences between social roles using a Fisher’s *post hoc* test (*p* < 0.05). Bars with differing letters indicate significant differences across treatments within individuals using a Fisher’s *post hoc* test.

Food sharing refusals were also associated with the parental status of the provisioner with the family, and food sharing was also affected by the interaction between parental status and nonapeptide treatment (Figure 3). Just as mothers shared more often than other caregivers, mothers also refused to share less often than any other caregivers [Figure 3, brackets; *F*_(1, 42.4)_ = 14.38, *p* < 0.001]. Fathers were the only family members whose rates of food sharing refusal were affected by nonapeptide treatment. Fathers treated with AVP had higher rates of food sharing refusals than when treated with Pro^8^-OT [Figure 3, letters; *F*_(2, 39.9)_ = 3.24, *p* = 0.050]. Recipient age did not affect food sharing refusals [*F*_(1, 33.04)_ = 2.64, *p* = 0.11].

**Figure 4:**
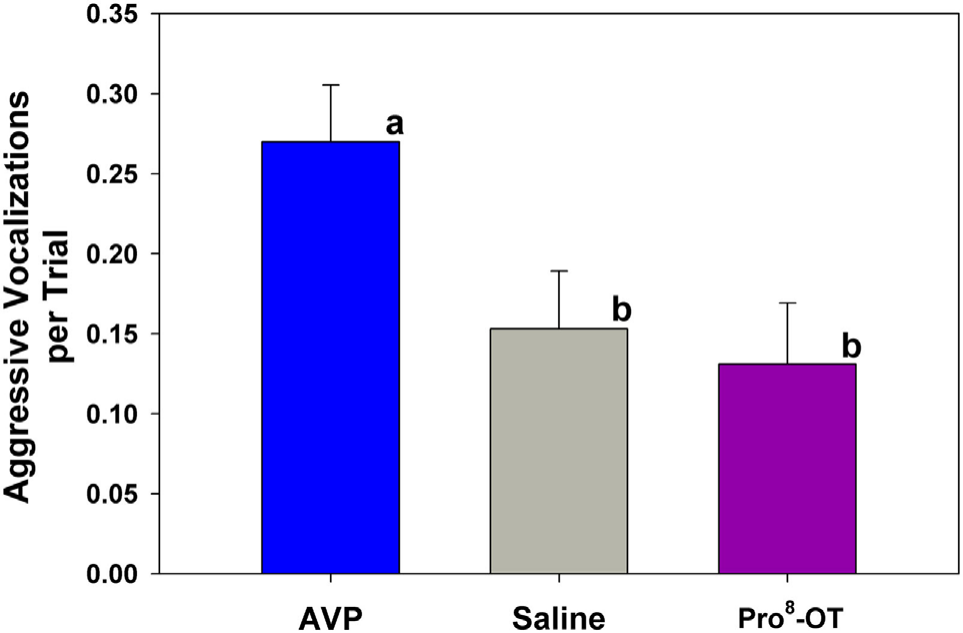
Aggressive vocalization emitted by caregivers (parents, alloparents). Treatment with arginine vasopressin (AVP) increased aggressive vocalizations compared to saline, Pro^8^-oxytocin (OT). Bars with differing letters indicate significant differences across treatments using a Fisher’s *post hoc* test.

Aggressive vocalizations (Ehr-Ehr) emitted by the provisioner during the food sharing test were associated with the parental status of the provisioner, as well as nonapeptide treatment. Alloparents emitted more aggressive vocalizations than parents did [*F*_(1, 694)_ = 13.52, *p* < 0.001; alloparents, M (± SEM) = 0.3 (0.03) vocalizations per trial; parents, M (± SEM) = 0.069 (0.05) vocalizations per trial]. Additionally, AVP increased aggressive vocalizations in provisioners compared to both Pro^8^-OT and saline in both parents and alloparents [Figure 4; *F*_(2, 694)_ = 4.49, *p* = 0.012]. There were no sex differences in provisioner aggressive vocalizations [*F*_(1, 694)_ = 0.02, *p* = 0.877], and sex did not interact with nonapeptide treatment or parental status [*F*’s < 0.31, *p*’s > 0.640]. Provisioners emitted marginally, but not significantly, more aggressive vocalizations toward younger recipients than they did toward older recipients [*F*_(1, 694)_ = 2.99, *p* = 0.08, Table S4 in Supplementary Material].

Finally, we evaluated whether nonapeptide treatment of provisioners altered rates of begging cries emitted by recipients. Nonapeptide treatment of the provisioner did not affect recipient begging cries, nor did parental status of the provisioner or the interaction (*F*’s < 2.24, *p* > 0.05). Begging cries did, however, vary by the recipient’s age; older recipients exhibited fewer begging cries than younger recipients [*b* = −0.0034, *F*_(1, 11.2)_ = 5.38, *p* = 0.040].

## Discussion

In marmosets, all family members perform post-weaning caregiving behavior in the form of food sharing, and we showed that nonapeptide treatment altered food sharing behavior in some, but not all, caregivers. Overall, mothers consistently provisioned food to recipients more frequently than fathers or alloparents did, and food provisioning behavior by mothers and female alloparents was not altered by manipulations of AVP or OT. The food provisioning behavior of male alloparents and fathers, however, was altered by AVP and OT treatment. Contrary to our hypothesis, AVP decreased provisioning behavior in fathers, and OT decreased provisioning behavior in male alloparents.

Previous studies in our lab indicated that AVP and OT enhance parental behavior and food sharing in marmosets. Food sharing among adults toward the pair mate was reduced following OTR *antagonist* treatment, suggesting that OT is important for prosocial food sharing behavior within the family (47). In a simulated infant distress paradigm, AVP enhanced responsiveness to infant stimuli in females, and OT enhanced responsiveness to infant stimuli in males (21), so we had expected to observe the same pattern with regard to food provisioning to juveniles; we expected AVP to enhance caregiving behavior in females, and OT to enhance caregiving behavior in males. However, in the context of maintaining monogamous pair bonds, previous work in our lab has provided evidence that OT may not always enhance prosocial behavior. OT did not enhance behavior directed toward the pair mate, but rather it reduced prosocial food sharing and sociosexual behavior directed toward opposite-sex strangers, thereby enhancing fidelity to the established pair mate (41, 43). Thus, AVP and OT may not enhance prosocial behavior generally, instead they may alter social decision-making based on context and social relationships.

We designed this experiment to expand upon the work done by Saito and Nakamura (20), who demonstrated that OT enhances food sharing in fathers toward younger (7–16 weeks), but not older (24–31 weeks) offspring. We expanded on the age range, and showed that OT does not alter paternal food sharing behavior toward older offspring (36–57 weeks). We used a different dose of OT and method of administration that Saito and Nakamura (20), and found no effect of OT on food sharing behavior toward older offspring in fathers. Escalating doses of OT and AVP produce differential behavioral effects in other species [e.g., Ref. (42, 48–50)] and it is likely that the same is true in marmosets. We used a single dose of each nonapeptide that affects adult pair-bonding behavior (41–43), but it may be the case that varying doses may have had differential behavioral effects in this context. With regard to fathers though, OT did not affect food sharing behavior at our intranasal dose or the i.c.v. dose used by Saito and Nakamura (20). An important distinction between our study and Saito and Nakamura’s (20) is that the OT ligand used differed: Saito and Nakamura used the conserved mammalian variant of OT (Leu^8^-OT), while we used the variant native to marmosets, Pro^8^-OT. Pro^8^-OT and Leu^8^-OT differentially affect marmoset social behavior in some contexts of adult pair bonds (41, 43, 51, 52). We also treated marmosets with AVP in our food sharing task, and compared to treatment with Pro^8^-OT. Like Saito and Nakamura, we found that OT at these doses did not affect paternal food sharing toward older offspring, and that AVP at this dose inhibited paternal food sharing behavior. We also included mothers and alloparents, and found that OT inhibited food sharing behavior in male alloparents. Our findings, combined with those of Saito and Nakamura, demonstrate that behavioral modification *via* AVP and OT is flexible; AVP- and OT-mediated food sharing behavior depends on multiple factors, including offspring age, caregiver sex, and parental status.

Food sharing behavior in primates is the product of multiple demographic and contextual variables. The relationship between OT and caregiving behavior in marmosets change with offspring age (20, 24), suggesting that OT modulates caregiving behavior dynamically with changing offspring and caregiver needs. Moreover, in large marmoset families, offspring age, caregiver experience, sex, and parental status interact to produce differential food provisioning behavior. Tolerance for food begging in adult marmosets wanes as offspring mature (53), reflecting reduced responsivity to signals for continued care from older offspring. Food sharing behavior in alloparents is also modulated by multiple variables, including sex and experience. Previous experience in rearing infants is associated with improved food sharing in male, but not female alloparents during undisturbed conditions (54). In an experimental task in which a response provided food to a younger family member, mothers, fathers, and male alloparents all selectively provided food to younger family members, but female alloparents exhibited lower scores on this measure (55). There is some evidence supporting the role of OT in altering social decision-making depending on social context, rather than enhancing global prosociality. In macaques, OT increases the willingness of male macaques to reward another macaque, but only when the alternative is to reward no one. However, when choosing to reward the self or another, OT increased selfish choices (56). In pair-bonded adult marmosets, OT does not increase food sharing with the pair mate, it instead decreases food sharing with an opposite-sex stranger (43). OT also reduces food sharing in group-housed adult capuchin monkeys, and it was suggested that this was mediated by OT-induced increases in social distance (57). It is likely that interactions between older and younger siblings, neither of which are wholly dependent on caregivers, will yield some selfish decision-making that is altered by hormonal neuromodulators like OT and AVP. Our findings speak to the broader issue of whether OT and AVP enhance prosocial behavior generally, or whether they alter social behavior depending on social context. We found that OT and AVP inhibited food sharing behavior, suggesting that OT and AVP alter social behavior depending on characteristics of the caregiver, rather than global enhancement of prosociality.

Arginine vasopressin is known to affect a wide range of aggressive behaviors, including maternal aggression [(3, 4, 10); c.f. (7, 8)], as well as territorial aggression [reviewed in Ref. (58)]. In general, the association between AVP and defense of offspring is limited to females (reviewed in Section “Introduction”), though not always (31), while AVP-mediated modulation of territorial aggression is often limited to males [reviewed in Ref. (58)]. We found that AVP increased aggressive vocalizations during food sharing trials, in males *and* females, as well as in parents and alloparents. There are two explanations for our lack of a sex effect. First, food aggression, maternal aggression, and territorial aggression may be controlled by different endocrine mechanisms, including AVP and OT. There is some evidence for this, as AVP V1b receptor knockout mice display impaired maternal and territorial aggression, but predatory aggression remains intact, suggesting that food aggression is different from defending offspring or territory (59, 60). However, while V1b knockout mice do compete for food, they do not compete as aggressively as wild types (59), weakening this argument. An alternative explanation for our lack of a sex effect in AVP-mediated aggression is that AVP and OT may affect aggressive behavior differently in primates than it does in rodents. There is some evidence for this, V1b receptor genetic polymorphisms human children are associated with aggression in both boys and girls, though they are more robust in boys than in girls (61, 62). Our findings highlight the need for more continued study of AVP, OT, and aggression in non-human primate models.

Oxytocin and AVP are involved in the modulation of dyadic interactions that are dependent on the behavior of both individuals. In humans, intranasal OT treatment in fathers enhances social reciprocity between father and infant, it also causes an increase in infant salivary OT and duration of social gaze (22). Similarly, high paternal plasma and salivary OT in human mothers and fathers is associated with father–infant coordination of affect (23, 63). Both AVP and OT are associated with dyadic interactions involving responding to infant gaze (9). This work in humans suggests that OT and AVP in the caregiver can affect behavior in the recipient. Previous work in our lab has shown that the behavior of an untreated marmoset is altered by OT treatment of the pair mate, suggesting that nonapeptides might alter the social attractiveness of a social partner (52). There is an important dyadic component to our measure of food sharing refusals. AVP-mediated increases in refusals may be the result of stable rates of begging and increased rates of refusal, or it may be the result of both increased rates of begging *and* increased refusal. However, begging cries emitted by the recipient were unaffected by nonapeptide treatment, suggesting that the behavior of recipients did not change in response to altered stimulus properties or any unobserved behavior of the caregiver.

There is considerable overlap between the OT and AVP systems in terms of neuroanatomical distributions [Reviewed in Ref. (64)] and receptor affinity [Reviewed in Ref. (6)], and there are also often important sex and species differences in the effects of OT and AVP on behavior. Given the considerable variation in NWM species OTRs and V1aRs, interactions between Pro^8^-OT and V1aR (or AVP and marmoset OTR) may be either reduced (i.e., greater receptor selectivity) or enhanced (i.e., greater receptor promiscuity) compared to humans, mice, and rats. Currently, the binding affinities and signaling potencies/efficacies of these ligand–receptor complexes is unknown. When AVP and OT are studied together, they provide valuable insights on these closely related systems, such as showing that OT and AVP act *via* one another’s receptors, and that they affect behavior synergistically. For example, both AVP and OT induce territorial marking in Syrian hamsters, but OT-induced marking is blocked by AVP receptor antagonists, not OTR antagonists (65). Similarly, blocking both OTRs and V1aRs reduced alloparental behavior in male voles, but blocking only one of these receptor types did not, indicating that AVP and OT work in concert to modulate male vole parental behavior (37). We found that AVP increased food sharing refusals in fathers, but not in male alloparents. Instead, for male alloparents, OT reduced total food sharing. These examples show that more information and nuance are gained from studying AVP and OT together than the sum of what is gained from studying each individually. These studies highlight the importance of comparing OT and AVP, especially in species with complex behavior and interindividual relationships.

## Ethics Statement

This study was carried out in accordance with the recommendations of the University of Nebraska Medical Center/University of Nebraska at Omaha Institutional Animal Care and Use Committee. The protocol was approved by the University of Nebraska Medical Center/University of Nebraska at Omaha Institutional Animal Care and Use Committee (protocol #15-005-04-FC).

## Author Contributions

JT, AI, and JF planned the experiment. JT and AI carried out the experiment under the supervision of JF. JT performed statistics, and JT, AI, and JF each contributed to writing and editing of the manuscript.

## Conflict of Interest Statement

The authors declare that the research was conducted in the absence of any commercial or financial relationships that could be construed as a potential conflict of interest.
