# Supplementary material for: Vasopressin and Oxytocin Reduce Food Sharing Behavior in Male, but Not Female Marmosets in Family Groups
Source: Front Endocrinol (Lausanne). 2017 Jul 27;8:181. doi: 10.3389/fendo.2017.00181 (PMC5529352; doi:10.3389/fendo.2017.00181)
Supplement: Supplementary file 1 [file table_1.docx]

Table S1.

|  | Variable | b | SS | MS | NumDF | DenDF | F | p |
| --- | --- | --- | --- | --- | --- | --- | --- | --- |
| Shares per Trial | Tx (AVP vs Pro8 vs SAL) |  | 0.0055 | 0.0027 | 2 | 26.11 | 0.0167 | 0.983413 |
|  | Sex (Male vs. Female) |  | 0.9645 | 0.9645 | 1 | 13.61 | 5.9156 | **0.029464** |
|  | Parent vs. alloparent |  | 0.0533 | 0.0533 | 1 | 11.59 | 0.3267 | 0.578512 |
|  | Food.Type (apple vs. cereal) |  | 0.696 | 0.696 | 1 | 671.17 | 4.2687 | **0.039203** |
|  | FamSize (N) | 3.9E-02 | 0.242 | 0.242 | 1 | 11.74 | 1.4845 | 0.247001 |
|  | InfantAge (days) | -1.43E-03 | 0.6157 | 0.6157 | 1 | 11.29 | 3.7765 | **0.077328** |
|  | Session.No | 2.37E-02 | 0.1448 | 0.1448 | 1 | 24.37 | 0.8884 | 0.355155 |
|  | Trial.No | 1.40 E-02 | 4.3489 | 4.3489 | 1 | 661.05 | 26.6745 | **3.19E-07** |
|  | Did.Not.See2 (y/n) |  | 1.5816 | 1.5816 | 1 | 678.67 | 9.7009 | **0.001919** |
|  | No.Interest2 (y/n) |  | 0.3593 | 0.3593 | 1 | 677.04 | 2.204 | 0.138115 |
|  | Watch2 (y/n) |  | 1.7874 | 1.7874 | 1 | 683.76 | 10.9632 | **0.000978** |
|  | Tx:Sex |  | 0.2731 | 0.1365 | 2 | 26.72 | 0.8375 | 0.443834 |
|  | Tx:Parent |  | 0.0992 | 0.0496 | 2 | 25.7 | 0.3042 | 0.740365 |
|  | Sex:Parent |  | 1.016 | 1.016 | 1 | 13.65 | 6.2315 | **0.026022** |
|  | Tx:Food.Type |  | 0.1714 | 0.0857 | 2 | 674.13 | 0.5256 | 0.591422 |
|  | Sex:Food.Type |  | 0.0776 | 0.0776 | 1 | 668.99 | 0.4759 | 0.490539 |
|  | Parent:Food.Type |  | 0.1295 | 0.1295 | 1 | 670 | 0.7941 | 0.373176 |
|  | Tx:Sex:Parent |  | 1.1238 | 0.5619 | 2 | 26.63 | 3.4466 | **0.046648** |
|  | Tx:Sex:Food.Type |  | 0.6213 | 0.3107 | 2 | 671.97 | 1.9054 | 0.149562 |
|  | Tx:Parent:Food.Type |  | 0.0316 | 0.0158 | 2 | 673.76 | 0.097 | 0.907533 |
|  | Sex:Parent:Food.Type |  | 1.4042 | 1.4042 | 1 | 669.2 | 8.6126 | **0.003453** |
|  | Tx:Sex:Parent:Food.Type |  | 0.0382 | 0.0191 | 2 | 672.45 | 0.117 | 0.889585 |

*Total Food shares per trial*

*Note:* Regression coefficients (b) provided for numerical variables only. Bolded p values were considered statistically significant (p<.05)

Table S2.

*Latency to share on each trial*

|  | Variable | b | SS | MS | NumDF | DenDF | F | p |
| --- | --- | --- | --- | --- | --- | --- | --- | --- |
| Latency to Share | Tx (AVP vs Pro8 vs SAL) |  | 29.3 | 14.7 | 2 | 25.44 | 0.039 | 0.961383 |
|  | Sex (Male vs. Female) |  | 2265.5 | 2265.5 | 1 | 13.65 | 6.098 | **0.027395** |
|  | Parent vs. alloparent |  | 117.5 | 117.5 | 1 | 11.63 | 0.316 | 0.584568 |
|  | Food.Type (apple vs. cereal) |  | 562.7 | 562.7 | 1 | 670.09 | 1.515 | 0.218881 |
|  | FamSize (N) | -1.55 | 383.7 | 383.7 | 1 | 11.78 | 1.033 | 0.329949 |
|  | InfantAge (days) | 0.073 | 1602.9 | 1602.9 | 1 | 11.31 | 4.314 | 0.061314 |
|  | Session.No | -0.89 | 176.6 | 176.6 | 1 | 23.92 | 0.475 | 0.497146 |
|  | Trial.No | -0.75 | 12596 | 12596 | 1 | 660.47 | 33.903 | **9.05E-09** |
|  | Did.Not.See2 (y/n) |  | 3232.8 | 3232.8 | 1 | 676.78 | 8.701 | **0.00329** |
|  | No.Interest2 (y/n) |  | 789.1 | 789.1 | 1 | 675.06 | 2.124 | 0.145478 |
|  | Watch2 (y/n) |  | 4560.4 | 4560.4 | 1 | 682.57 | 12.275 | **0.000489** |
|  | Tx:Sex |  | 303.2 | 151.6 | 2 | 26.19 | 0.408 | 0.669067 |
|  | Tx:Parent |  | 219.5 | 109.8 | 2 | 25.09 | 0.295 | 0.746781 |
|  | Sex:Parent |  | 2705.3 | 2705.3 | 1 | 13.7 | 7.282 | **0.017578** |
|  | Tx:Food.Type |  | 378.1 | 189 | 2 | 672.97 | 0.509 | 0.601444 |
|  | Sex:Food.Type |  | 330.3 | 330.3 | 1 | 668.08 | 0.889 | 0.346099 |
|  | Parent:Food.Type |  | 32.7 | 32.7 | 1 | 668.95 | 0.088 | 0.766674 |
|  | Tx:Sex:Parent |  | 2252.7 | 1126.3 | 2 | 26.1 | 3.032 | 0.065479 |
|  | Tx:Sex:Food.Type |  | 1713.4 | 856.7 | 2 | 670.96 | 2.306 | 0.100459 |
|  | Tx:Parent:Food.Type |  | 414.9 | 207.5 | 2 | 672.59 | 0.558 | 0.572379 |
|  | Sex:Parent:Food.Type |  | 1679.5 | 1679.5 | 1 | 668.28 | 4.52 | **0.033858** |
|  | Tx:Sex:Parent:Food.Type |  | 498.1 | 249.1 | 2 | 671.45 | 0.67 | 0.511858 |

*Note:* Regression coefficients (b) provided for numerical variables only. Bolded p values were considered statistically significant (p<.05)

Table S3.

*Begs with refusal per trial*

|  | Variable | b | SS | MS | NumDF | DenDF | F | p |
| --- | --- | --- | --- | --- | --- | --- | --- | --- |
| Beg with refusal | Tx (AVP vs Pro8 vs SAL) |  | 1.452 | 0.726 | 2 | 39.5 | 1.3581 | 0.268897 |
|  | Sex (Male vs. Female) |  | 13.306 | 13.306 | 1 | 41.36 | 24.8914 | **1.14E-05** |
|  | Parent vs. alloparent |  | 3.4917 | 3.4917 | 1 | 39.09 | 6.5318 | **0.014597** |
|  | Food.Type (apple vs. cereal) |  | 3.2407 | 3.2407 | 1 | 676.04 | 6.0623 | **0.014058** |
|  | FamSize (N) | 3.41E-02 | 1.2514 | 1.2514 | 1 | 43.59 | 2.3409 | 0.133241 |
|  | InfantAge (days) | -7.83E-04 | 1.4102 | 1.4102 | 1 | 33.04 | 2.6381 | 0.113834 |
|  | Session.No | 1.16e-02 | 0.0415 | 0.0415 | 1 | 35.83 | 0.0775 | 0.782259 |
|  | Trial.No | -4.55E-03 | 0.4608 | 0.4608 | 1 | 663.74 | 0.862 | 0.353503 |
|  | Did.Not.See2 (y/n) |  | 3.0083 | 3.0083 | 1 | 689.43 | 5.6276 | **0.017954** |
|  | No.Interest2 (y/n) |  | 8.5729 | 8.5729 | 1 | 688.45 | 16.0371 | **6.89E-05** |
|  | Watch2 (y/n) |  | 13.0921 | 13.0921 | 1 | 693.8 | 24.4912 | **9.38E-07** |
|  | Tx:Sex |  | 1.1254 | 0.5627 | 2 | 39.86 | 1.0526 | 0.358523 |
|  | Tx:Parent |  | 0.5297 | 0.2648 | 2 | 38.8 | 0.4954 | 0.613116 |
|  | Sex:Parent |  | 7.689 | 7.689 | 1 | 42.4 | 14.3838 | **0.000467** |
|  | Tx:Food.Type |  | 0.6675 | 0.3337 | 2 | 678.09 | 0.6243 | 0.535933 |
|  | Sex:Food.Type |  | 0.4743 | 0.4743 | 1 | 672.2 | 0.8873 | 0.346555 |
|  | Parent:Food.Type |  | 0.2758 | 0.2758 | 1 | 674.58 | 0.516 | 0.472793 |
|  | Tx:Sex:Parent |  | 3.463 | 1.7315 | 2 | 39.88 | 3.2391 | **0.049715** |
|  | Tx:Sex:Food.Type |  | 0.8205 | 0.4102 | 2 | 674.31 | 0.7674 | 0.464617 |
|  | Tx:Parent:Food.Type |  | 0.0038 | 0.0019 | 2 | 677.49 | 0.0035 | 0.996464 |
|  | Sex:Parent:Food.Type |  | 1.4777 | 1.4777 | 1 | 672.07 | 2.7644 | 0.09685 |
|  | Tx:Sex:Parent:Food.Type |  | 0.3363 | 0.1681 | 2 | 675.06 | 0.3145 | 0.730235 |

*Note:* Regression coefficients (b) provided for numerical variables only. Bolded p values were considered statistically significant (p<.05)

Table S4.

*Aggressive vocalizations per trial*

|  | Variable | b | SS | MS | NumDF | DenDF | F | p |
| --- | --- | --- | --- | --- | --- | --- | --- | --- |
| Aggressive Vocalizations | Tx (AVP vs Pro8 vs SAL) |  | 2.1336 | 1.0668 | 2 | 694 | 4.4937 | **0.011506** |
|  | Sex (Male vs. Female) |  | 0.0056 | 0.0056 | 1 | 694 | 0.0237 | 0.877653 |
|  | Parent vs. alloparent |  | 3.2085 | 3.2085 | 1 | 694 | 13.5152 | **0.000255** |
|  | Food.Type (apple vs. cereal) |  | 0.317 | 0.317 | 1 | 694 | 1.3351 | 0.248297 |
|  | FamSize (N) | -1.54E-02 | 0.286 | 0.286 | 1 | 694 | 1.2047 | 0.272769 |
|  | InfantAge (days) | -5.18E-04 | 0.7099 | 0.7099 | 1 | 694 | 2.9903 | 0.084211 |
|  | Session.No | 1.70e-02 | 0.102 | 0.102 | 1 | 694 | 0.4298 | 0.512301 |
|  | Trial.No | -9.22E-03 | 1.8974 | 1.8974 | 1 | 694 | 7.9924 | **0.004833** |
|  | Did.Not.See2 (y/n) |  | 0.4576 | 0.4576 | 1 | 694 | 1.9277 | 0.165456 |
|  | No.Interest2 (y/n) |  | 0.2781 | 0.2781 | 1 | 694 | 1.1714 | 0.27948 |
|  | Watch2 (y/n) |  | 0.4843 | 0.4843 | 1 | 694 | 2.04 | 0.153658 |
|  | Tx:Sex |  | 0.1458 | 0.0729 | 2 | 694 | 0.3071 | 0.735677 |
|  | Tx:Parent |  | 0.3181 | 0.159 | 2 | 694 | 0.6699 | 0.512114 |
|  | Sex:Parent |  | 0.0521 | 0.0521 | 1 | 694 | 0.2195 | 0.639545 |
|  | Tx:Food.Type |  | 0.2682 | 0.1341 | 2 | 694 | 0.5648 | 0.568744 |
|  | Sex:Food.Type |  | 0.2826 | 0.2826 | 1 | 694 | 1.1904 | 0.275627 |
|  | Parent:Food.Type |  | 1.6623 | 1.6623 | 1 | 694 | 7.0019 | **0.008327** |
|  | Tx:Sex:Parent |  | 0.5625 | 0.2812 | 2 | 694 | 1.1846 | 0.306486 |
|  | Tx:Sex:Food.Type |  | 0.0835 | 0.0418 | 2 | 694 | 0.1759 | 0.838711 |
|  | Tx:Parent:Food.Type |  | 0.2354 | 0.1177 | 2 | 694 | 0.4958 | 0.609304 |
|  | Sex:Parent:Food.Type |  | 0.3544 | 0.3544 | 1 | 694 | 1.4928 | 0.222194 |
|  | Tx:Sex:Parent:Food.Type |  | 0.0461 | 0.0231 | 2 | 694 | 0.0971 | 0.907454 |

*Note:* Regression coefficients (b) provided for numerical variables only. Bolded p values were considered statistically significant (p<.05)

Table S5.

*Recipient begging cries per trial*

|  | Variable | b | SS | MS | NumDF | DenDF | F | p |
| --- | --- | --- | --- | --- | --- | --- | --- | --- |
| Recipient Begging Cries | Tx (AVP vs Pro8 vs SAL) |  | 1.8314 | 0.9157 | 2 | 25.02 | 2.2419 | 0.12717 |
|  | Sex (Male vs. Female) |  | 0.4408 | 0.4408 | 1 | 14.24 | 1.0791 | 0.31621 |
|  | Parent vs. alloparent |  | 0.3989 | 0.3989 | 1 | 11.37 | 0.9767 | 0.34359 |
|  | Food.Type (apple vs. cereal) |  | 7.7699 | 7.7699 | 1 | 665.06 | 19.0232 | **1.50E-05** |
|  | FamSize (N) | 0.031 | 0.0975 | 0.0975 | 1 | 11.45 | 0.2387 | 0.63438 |
|  | InfantAge (days) | -0.0034 | 2.1991 | 2.1991 | 1 | 11.16 | 5.384 | **0.04024** |
|  | Session.No | -0.0045 | 0.0021 | 0.0021 | 1 | 24.43 | 0.005 | 0.94406 |
|  | Trial.No | 0.0054 | 0.6474 | 0.6474 | 1 | 659.37 | 1.585 | 0.20849 |
|  | Did.Not.See2 (y/n) | -0.21 | 1.3816 | 1.3816 | 1 | 667.88 | 3.3827 | 0.06633 |
|  | No.Interest2 (y/n) |  | 1.3843 | 1.3843 | 1 | 666.57 | 3.3893 | 0.06606 |
|  | Watch2 (y/n) |  | 1.6026 | 1.6026 | 1 | 672.67 | 3.9236 | **0.04802** |
|  | Tx:Sex |  | 0.3208 | 0.1604 | 2 | 26.56 | 0.3927 | 0.67911 |
|  | Tx:Parent |  | 1.5484 | 0.7742 | 2 | 24.85 | 1.8955 | 0.17133 |
|  | Sex:Parent |  | 1.0987 | 1.0987 | 1 | 14.26 | 2.69 | 0.12285 |
|  | Tx:Food.Type |  | 0.4729 | 0.2364 | 2 | 667.02 | 0.5788 | 0.56083 |
|  | Sex:Food.Type |  | 0.0007 | 0.0007 | 1 | 664.64 | 0.0016 | 0.96775 |
|  | Parent:Food.Type |  | 0.3888 | 0.3888 | 1 | 664.31 | 0.9519 | 0.3296 |
|  | Tx:Sex:Parent |  | 0.8243 | 0.4122 | 2 | 26.55 | 1.0091 | 0.3781 |
|  | Tx:Sex:Food.Type |  | 0.4988 | 0.2494 | 2 | 666.48 | 0.6106 | 0.54332 |
|  | Tx:Parent:Food.Type |  | 0.9208 | 0.4604 | 2 | 666.74 | 1.1272 | 0.32454 |
|  | Sex:Parent:Food.Type |  | 0.9384 | 0.9384 | 1 | 664.75 | 2.2975 | 0.13005 |
|  | Tx:Sex:Parent:Food.Type |  | 0.0457 | 0.0229 | 2 | 666.9 | 0.056 | 0.94557 |

*Note:* Regression coefficients (b) provided for numerical variables only. Bolded p values were considered statistically significant (p<.05)
